# Sodium Rich Vanadium Oxy‐Fluorophosphate – Na_3.2_Ni_0.2_V_1.8_(PO_4_)_2_F_2_O – as Advanced Cathode for Sodium Ion Batteries

**DOI:** 10.1002/advs.202301091

**Published:** 2023-05-18

**Authors:** Rachid Essehli, Hamdi Ben Yahia, Ruhul Amin, Mengya Li, Daniel Morales, Steven G. Greenbaum, Ali Abouimrane, Anand Parejiya, Abdelfattah Mahmoud, Khalid Boulahya, Marm Dixit, Ilias Belharouak

**Affiliations:** ^1^ Electrification and Energy Infrastructures Division Oak Ridge National Laboratory Oak Ridge TN 37831 USA; ^2^ Qatar Environment and Energy Research Institute Hamad Bin Khalifa University Qatar Foundation Doha 34110 Qatar; ^3^ Exponent, Inc. Natick MA 01760 USA; ^4^ Department of Physics & Astronomy Hunter College of the City University of New York New York NY 10065 USA; ^5^ Greenmat Cesam Research Unit University of Liège Department of Chemistry Liège 4000 Belgium; ^6^ Departamento de Química Inorgánica Facultad de Químicas Universidad Complutense Madrid 28040 Spain

**Keywords:** energy storage, high‐voltage cathode, in situ X‐ray, sodium‐ion battery, vanadium Oxy‐fluorophosphate

## Abstract

Conventional sodium‐based layered oxide cathodes are extremely air sensitive and possess poor electrochemical performance along with safety concerns when operating at high voltage. The polyanion phosphate, Na_3_V_2_(PO_4_)_3_ stands out as an excellent candidate due to its high nominal voltage, ambient air stability, and long cycle life. The caveat is that Na_3_V_2_(PO_4_)_3_ can only exhibit reversible capacities in the range of 100 mAh g^−1^, 20% below its theoretical capacity. Here, the synthesis and characterizations are reported for the first time of the sodium‐rich vanadium oxyfluorophosphate, Na_3.2_Ni_0.2_V_1.8_(PO_4_)_2_F_2_O, a tailored derivative compound of Na_3_V_2_(PO_4_)_3_, with extensive electrochemical and structural analyses. Na_3.2_Ni_0.2_V_1.8_(PO_4_)_2_F_2_O delivers an initial reversible capacity of 117 mAh g^−1^ between 2.5 and 4.5 V under the 1C rate at room temperature, with 85% capacity retention after 900 cycles. The cycling stability is further improved when the material is cycled at 50 °C within 2.8–4.3 V for 100 cycles. When paired with a presodiated hard carbon, Na_3.2_Ni_0.2_V_1.8_(PO_4_)_2_F_2_O cycled with a capacity retention of 85% after 500 cycles. Cosubstitution of the transition metal and fluorine in Na_3.2_Ni_0.2_V_1.8_(PO_4_)_2_F_2_O as well as the sodium‐rich structure are the major factors behind the improvement of specific capacity and cycling stability, which paves the way for this cathode in sodium‐ion batteries.

## Graphical abstract

This work reports Na_3.2_Ni_0.2_V_1.8_(PO_4_)_2_F_2_O, a tailored derivative compound of Na_3_V_2_(PO_4_)_3_, with extensive electrochemical and structural analyses. The developed material shows an initial reversible capacity of 117 mAh g^−1^ with 91% retention over 900 cycles. Sodium rich structure and cosubstitution are identified to be the drivers of improvements in specific capacity and durability of the cathode material.

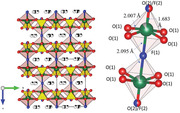

## 1 Introduction

Lithium‐ion batteries (LIBs) are currently the preferred energy storage technology for most electronic devices and electric vehicles. However, the pressing demand on lithium will likely be a hurdle to support the widespread of LIBs use in electric vehicle, utility grids, and a range of present and future applications.^[^ ^1^ ^]^ Thus, the development of alternative technologies to LIBs is of utmost significance for the deployment of large scale energy storage technologies. Several studies anticipated that the use of sodium‐ion batteries with renewable energies would significantly lower the levelized cost of electricity.^[^ ^2^ ^]^ Indeed, despite the low energy density penalty, sodium‐ion batteries are viewed as attractive alternatives because of (1) abundance of sodium, (2) use of less critical components, (3) the ability to leverage and use the manufacturing knowhow of LIBs.^[^ ^3^ ^]^

NaVPO_4_F,^[^ ^4^ ^]^ Na_3_V_2_(PO_4_)_2_F_3−_ *_x_* O*_x_*,^[^ ^5^ ^]^ Na_4_MnV(PO_4_)_3_,^[^ ^6^ ^]^ Na_2_Ni_2_Fe(PO_4_)_3_,^[^ ^7^ ^]^ and Na_4_Fe_3_(PO_4_)_2_(P_2_O_7_),^[^ ^8^ ^]^ are example of the most studied sodium‐based polyanion cathode materials. To improve the electrochemical performance of Na_3_V_2_(PO_4_)_3_ (NVP), Li et al.^[^ ^9^ ^]^ prepared a series of sodium‐rich compounds, Na_3+_ *_x_* V_2−_ *_x_* M*_x_* (PO_4_)_3_ (M = Ni, Mg), by a sol–gel synthesis and examined the impact of Ni^2+^ and Mg^2+^ substitutions on its crystal structure in a correlation with its electrochemical performance. Bag et al.^[^ ^10^ ^]^ found that the introduction of Mg^2+^ into Na_3+_ *_x_* V_2−_ *_x_* M*_x_* (PO_4_)_3_ (M =nickel, cobalt, magnesium) led to a drastic improvement of the rate performance and cycling of the NVP cathode. Zhang et al.^[^ ^11^ ^]^ used a hydrothermal method to synthesize spherical Na_3_V_1.95_Mn_0.05_(PO_4_)_2_F_3_. The researchers suggested that the Mn‐doping increased the unit cell volume and facilitated sodium ion transport during the charge and discharge processes. To compensate for the sodium loss during the formation of solid electrolyte interphase (SEI), Zhang et al.^[^ ^12^ ^]^ synthesized a sodium‐rich and fluorinated phosphate, Na_4_V_2_(PO_4_)_2_F_3_, using a mechanical milling of Na_3_V_2_(PO_4_)_2_F_3_ and sodium metal. Bianchini et al.^[^ ^13^ ^]^ synthesized Na_4_V_2_(PO_4_)_2_FO_2_ by the electrochemical insertion of one sodium into Na_3_V_2_(PO_4_)_2_FO_2_.

Our research team developed several advanced sodium‐based cathodes such as Na_4_MnV(PO_4_)_3_,^[^ ^3^, ^6^ ^]^ NaFe_2−_ *_x_* V*_x_* (PO_4_)(SO_4_)_2_ (0 ≤ *x* ≤ 1),^[^ ^14^ ^]^ Na_2_M_2_Fe(PO_4_)_3_ (M = Co, Ni, Fe),^[^ ^7^ ^]^ Na_4_Ni_3_(PO_4_)_2_(P_2_O_7_),^[^ ^8^ ^]^ and Na*_x_* Ni*_y_* Mn_1−_ *_y_* O_2_..^[^ ^15^ ^]^ We also synthesized Na_3_V_1.7_Fe_0.3_O(PO_4_)_2_F_2_^[^ ^16^ ^]^ and Na_3_V_2_(PO_4_)_2_F_3_^[^ ^17^ ^]^ using a hydrothermal‐assisted sol–gel method. These materials exhibited outstanding electrochemical performance in half‐ and full‐cell configurations at high temperatures (45 and 55 °C). Herein, we report for the first time a hydrothermal synthesis of a new class of sodium‐rich cathode material with the formula Na_3.2_Ni_0.2_V_1.8_(PO_4_)_2_F_2_O that could enable the compensation of the irreversible sodium loss in the first charge in a full cell with presodiated hard carbon. The crystal structure was refined by the Rietveld method using high‐resolution synchrotron powder diffraction data and was confirmed by high‐resolution transmission electron microscopy. The phase stability during cycling was also analyzed using in situ X‐ray powder diffraction (XRD). The electrochemical performances were evaluated in sodium half cells at room temperature and at 50 °C by galvanostatic cycling, cyclic voltammetry, and electrochemical impedance spectroscopy, whereas the full cells were evaluated at room temperature. The Na_3.2_Ni_0.2_V_1.8_(PO_4_)_2_F_2_O cathode material demonstrates a capacity retention of 85% after 900 cycles in half cell at room temperature. To our knowledge, no similar compounds with other alkali ion have been reported so far in the literature.

## 2 Results and discussion

### 2.1 Structure Refinement

**Figure 1:**
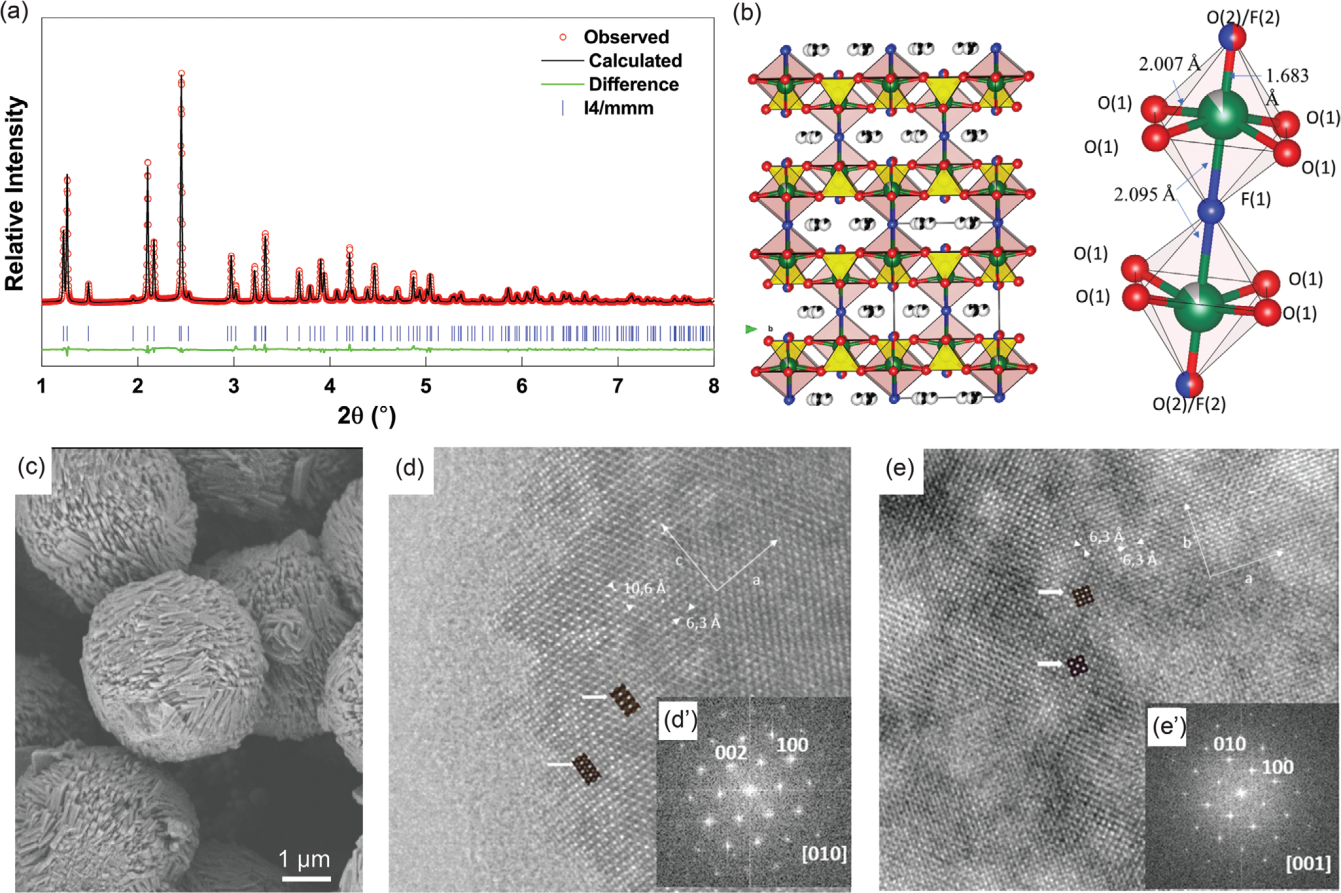
a) Observed, calculated, and difference plots for X‐ray powder diffraction (*λ* = 0.1173 Å) refinement of Na_3.2_Ni_0.2_V_1.8_(PO_4_)_2_F_2_O powder. b) Crystal structure representation of Na_3.2_Ni_0.2_V_1.8_(PO_4_)_2_F_2_O, with VO_5_F octahedra and PO_4_ tetrahedra depicted as yellow polyhedral and Na^+^ ions represented by black spheres and the schematic of the bioctahedra (V/Ni)_2_O_10_F_2_. c) SEM image of the synthesized powder. A high‐resolution transmission electron microscopy image of Na_3.2_Ni_0.2_V_1.8_(PO_4_)_2_F_2_O along d) [010] and e) [001] zone axis and corresponding Fast Fourier transform (inset of [d]) and (inset of [e]), respectively. Calculated images are shown in the insets and marked by white arrows.

The synthesized cathode material Na_3.2_Ni_0.2_V_1.8_(PO_4_)_2_F_2_O was analyzed using high‐resolution synchrotron XRD. The diffractogram of the material recorded in the 2*θ* range of 1–10 Å (*λ* = 0.1173 Å) is illustrated in **Figure** **1**a. The XRD pattern showed no impurities, and all the peaks are sharp and well‐defined, which indicates a high degree of crystallinity of the Na_3.2_Ni_0.2_V_1.8_(PO_4_)_2_F_2_O powder.

The crystal structure of Na_3.2_Ni_0.2_V_1.8_(PO_4_)_2_F_2_O was refined using the structure of Na_3_V_2_(PO_4_)_2_F_3_ (space group = I4/mmm) as a starting model (Table S1, Supporting Information).^[^ ^18^ ^]^ At first, a statistical disorder of Ni/V was introduced in the vanadium site, and constraints on their occupancies and atomic displacement parameters (ADPs) were applied. The occupancies of the sodium atoms were then refined, showing a slight increase for Na(1) at the 8 *h* site and no changes for Na(2) content at the 16*l* site. This led to a composition close to Na_3.2_Ni_0.2_V_1.8_(PO_4_)_2_F_3_. The presence of vanadyl (V = O) bonds was confirmed by IR absorption spectroscopy. The presence of a vibration mode at ≈911 cm^−1^ is in good agreement with previous studies on Na_3_V_2_O_2_ *_x_* (PO_4_)_2_F_3−2_ *_x_* ^[^ ^19^ ^]^ (Figure S1, Supporting Information). Therefore, a statistical disorder of F(2)/O(2) was introduced at the F(2) site, and constraints on their occupancies and ADPs were applied. The Rietveld analysis led to the reliability factors listed in **Table** **1** (*R*_p_ = 0.0448, *R*_wp_ = 0.0605, *R*_B_ = 0.0493, and *χ* ^2^ = 3.03) and the composition of Na_3.2_Ni_0.2_V_1.8_(PO_4_)_2_F_2_O. The final atomic positions are given in **Table** **2**. Figure 1a shows an excellent agreement between the experimental and calculated patterns.

**Table 1:** Crystallographic data and structure refinement for Na_3.2_Ni_0.2_V_1.8_(PO_4_)_2_F_2_O

**Crystal data**	
Chemical formula	Na_3.2_Ni_0.2_V_1.8_(PO_4_)_2_F_2_O
Molecular Weight (M.W.)	420.9
Space group	I4/mmm
Temperature [K]	293
*a* [Å]	6.3939(1)
*c* [Å]	10.6326(2)
*V* [Å^3^]	434.68(1)
*Z*	2

**Refinement**	
*R*_p_	0.0448
*R*_wp_	0.0605
*R*_B_	0.0493
G.O.F. *χ*2	3.03
No. of parameters	36
No. of constraints	4

**Table 2:** Atom coordinates and isotropic atomic displacement parameters (Å^2^) for Na_3.2_Ni_0.2_V_1.8_(PO_4_)_2_F_2_O

Atom	Wyck.	Occ.	*x*	*y*	*z*	Uiso [Å^2^]
Na1	8h	0.5	0.2634(5)	0.2634(5)	0	0.013(1)
Na2	16l	0.15	0.3536(16)	0.2214(14)	0	0.052(3)
V1/Ni1	4e	0.9/0.1	0	0	0.19705(9)	0.0096(3)
P1	4d	1	1/2	0	1/4	0.0052(4)
O1	16n	1	0.3087(2)	0	0.16311(12)	0.0037(4)
F1	2a	1	0	0	0	0.0049(1)
F2/O2	4e	0.5/0.5	0	0	0.3553(4)	0.0262(1)

The structure of Na_3.2_Ni_0.2_V_1.8_(PO_4_)_2_F_2_O can be described as a 3D network of bioctahedra (V/Ni)_2_O_10_F_2_ that are connected through the PO_4_ tetrahedra (Figure 1b). The Na^+^ cations are in the tunnels running along the three main crystallographic directions, which are [100], [010], and [001]. The (V/Ni)O_5_F octahedra share a common fluorine corner (Figure 1b). The octahedra (V/Ni)O_5_F consists of four equivalent V–O(1) bond distances of 2.007 Å, as well as one short bond distance (V/Ni) = O(2) and one stretched bond distance (V/Ni)–F(1) of 1.683(1) and 2.095(1) Å, respectively. These distances are in good agreement with those observed in the polyanionic compounds Na_3_(VO)Fe(PO_4_)_2_F_2_, Na_3_VO(PO_4_)_2_F, and Na_3_V_2_(PO_4_)_2_F_3_ with 1.65 and 1.934 Å for the short and stretched distances, respectively.^[^ ^20^ ^]^ The PO_4_ tetrahedra have four P–O(1) bonds with distance of 1.533 Å^[^ ^21^ ^]^ which is similar to the P–O distances observed in most of the phosphate materials ^[^ ^20^ ^]^ Besides the chemical compositions, the main difference between Na_3.2_Ni_0.2_V_1.8_(PO_4_)_2_F_2_O and Na_3_V_2_(PO_4_)_2_F_3_ consists in the shift along the c axis of the V atoms, from the basal plan of the octahedra towards the F2/O2 atoms in order to form the vanadyl (V = O) bond. One should notice that V, F1 and F2/O2 atoms are aligned along the c axis (Figure 1b).

### 2.2 Electron Microscopy Results

The scanning electron microscopy (SEM) image (Figure 1c) indicate that Na_3.2_Ni_0.2_V_1.8_(PO_4_)_2_F_2_O has high‐quality monodispersed particles with a homogeneous spherical morphology and an average particle size of ≈11 µm. Further, the EDS analysis confirms the nominal composition expected of the synthesized materials (Table S2, Supporting Information). According to the Na/V ratio from the EDX result, it indicates that Ni2+ goes to a V site and excess Na+ was introduce into the crystal to keep the charge balance. To further confirm the X‐ray structural model, high resolution transmission electron microscopy (HRTEM) studies were performed along the most informative orientations, which in this case were along [010] and [001] (Figure 1d,e). The composition, determined on several small crystallites by energy‐dispersive X‐ray analysis in the electron microscope, agrees with the nominal one, except for light elements, although their presence is confirmed. The corresponding HRTEM micrograph (Figure 1d) along [010] shows an apparently well‐ordered material with *d*‐spacings of 6.3 and 10.6 Å, corresponding to d_100_ and d_001_, respectively. Fast Fourier transform was performed on the HRTEM micrograph (inset images in Figure 1d,e), indicating the existence of different domains. However, the whole crystal results were clearly homogeneous, and only the maxima corresponding to the plane (*ac*) of the centered tetragonal unit cell were observed. The HRTEM image along [001] (Figure 1e) clearly confirms the tetragonal centered unit cell because well‐ordered material with *d*‐spacings of 6.3 and 6.3 Å corresponding to d_100_ and d_010_ are observed. Fast Fourier transform performed on the HRTEM image (inset in Figure 1e) shows that we are dealing with the basal plane (*ab*) of the tetragonal centered unit cell. Calculated images using a tetragonal centered unit cell fit nicely in this orientation with the experimental image in both thin and thick parts of the crystal. Based on the above results, the information obtained from HRTEM images confirm that the crystal structure of Na_3.2_Ni_0.2_V_1.8_(PO_4_)_2_F_2_O corresponds to tetragonal centered unit cell (space group = I4/mmm) as refined by using high‐resolution synchrotron XRD.

### 2.3 NMR Studies

#### 2.3.1 ^19^F NMR

The NMR measurements indicate that two peaks exist, located at −168 and −203 ppm, suggesting fluorine is in two different chemical environments. The ^19^F spectrum for Na_3.2_Ni_0.2_V_1.8_(PO_4_)_2_F_2_O is displayed in Figure S2 in the Supporting Information. Assuming the crystal structure of the Na_3.2_Ni_0.2_V_1.8_(PO_4_)_2_F_2_O is similar to that of pristine Na_3_V_2_(PO_4_)_2_F_3_, there are two known fluorine sites in the material—F(1) and F(2). The peak at −203 ppm corresponds to the fluorine in the F(2) site, owing to bonding with more Na sites than the F(1) sites and shorter bond length.^[^ ^18^ ^]^

#### 2.3.2 ^31^P NMR

The NMR spectra for ^31^P in Na_3.2_Ni_0.2_V_1.8_(PO_4_)_2_F_2_O are shown in Figures S3 and S4 in the Supporting Information. Because of the inclusion of paramagnetic vanadium and nickel, there is considerable anisotropy in the ^31^P environment, denoted by the strong presence of spinning sidebands past 20 kHz. Three clear peaks are visible at 11, −11, and −156 ppm. In addition, there exists a considerably broad region from 1000 to 4000 ppm. Because of the limited excitation bandwidth of NMR pulses, this region was detected using a stepwise spin‐echo method.^[^ ^22^ ^]^ This region could be due to the presence of V^4+^ oxidation states in the material.

#### 2.3.3 ^23^Na NMR

The sodium NMR of the Na_3.2_Ni_0.2_V_1.8_(PO_4_)_2_F_2_O exhibits three peaks, located at 127, 69, and 58 ppm (Figure S5, Supporting Information). This is in contrast to previous studies on pristine Na_3_V_2_(PO_4_)_2_F_3_, which is known to exhibit two major peaks at 92 and 146 ppm.^[^ ^23^ ^]^ While the two downfield peaks may have shifted upfield due to the inclusion of nickel, the upfield peak at 58 ppm does not correspond to any known 23Na peaks in the pristine material. It should be noted that the NMR is much more sensitive than the XRD; thus, the third site may correspond to an amorphous phase that would not be detected by XRD.

### 2.4 Electrochemical Performance

**Figure 2:**
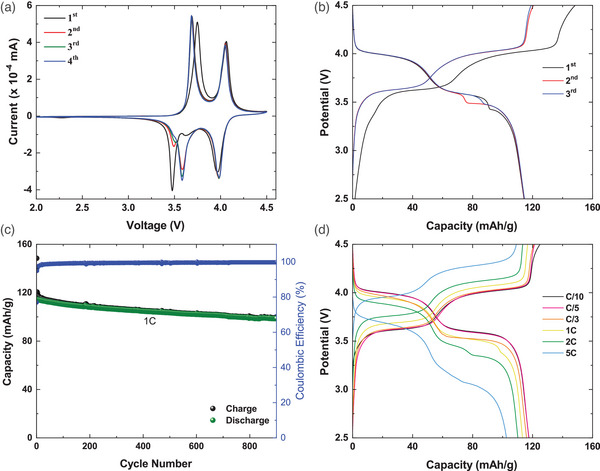
a) Cyclic voltammogram curves recorded at room temperature, between 2 and 4.5 V versus Na^+^/Na, with a scanning rate of 0.05 mV s^−1^. b) Charge/discharge curves of Na_3.2_Ni_0.2_V_1.8_(PO_4_)_2_F_2_O sample, recorded at room temperature, in a CC mode, at 1C rate, in the voltage range 2.5–4.5 V versus Na^+^/Na. c) Cycling performance of Na_3.2_Ni_0.2_V_1.8_(PO_4_)_2_F_2_O half‐cell at 1C recorded room temperature. d) Charge/discharge curves of Na_3.2_Ni_0.2_V_1.8_(PO_4_)_2_F_2_O at C/10, C/5, C/3, 1C, 2C, and 5C rates between 2.5 and 4.5 V versus Na^+^/Na.

**Figure** **2**a shows the cyclic voltammetry (CV) curves of Na_3.2_Ni_0.2_V_1.8_ (PO_4_)_2_F_2_O for four cycles recorded at 0.05 mV s^−1^ in the voltage window of 2–4.5 V versus Na^+^/Na. The cyclic voltammetry tests indicate the presence of two reversible peaks at 3.6 and 4.1 V, which lead to an average operation voltage of 3.85 V. The first redox couple corresponding to a V^3+^/V^4+^ redox has anodic and cathodic peaks centered at around 3.7 and 3.6 V respectively. This corresponds to the reversible extraction and insertion of one sodium atom into the crystal structure of Na_3.2_Ni_0.2_V_1.8_(PO_4_)_2_F_2_O. The second redox couple at 4.0 and 3.9 V corresponds to the V^4+^/V^5+^ and the reversible intercalation of a second sodium atom. These two redox couples enabled extraction/insertion of two sodium atoms associated with V^3+/^V^4+^ and V^4+^/V^5+^ consistent with those reported in the literature for Na_3_(VO)_1.7_Fe_0.3_(PO_4_)_2_F_1.3_, Na_3_V_2_O_1.6_(PO_4_)_2_F_1.4_, and Na_3_(VO_0.5_)_2_(PO_4_)_2_F_2_ cathode materials. Additionally, Na_3.2_Ni_0.2_V_1.8_(PO_4_)_2_F_2_O material operates at an average voltage of ≈3.85 V, which is higher than those reported for cathodes NaFe_2_(SO_4_)_2_(PO_4_) (2.8 V),^[^ ^14a^ ^]^ Na_1.86_Fe_3_(PO_4_)_3_ (3 V),^[^ ^7c,d^ ^]^ and Na_4_MnV(PO_4_)_3_ (3.2 V)^[^ ^3^, ^6^ ^]^

Figure 2b presents the galvanostatic charge/discharge curves of Na_3.2_Ni_0.2_V_1.8_(PO_4_)_2_F_2_O in sodium half‐cell during the first three cycles. The curves show that the Na_3.2_Ni_0.2_V_1.8_(PO_4_)_2_F_2_O| Na half‐cell exhibits two potential plateaus at 4.02/4.25 and 3.74/3.96 V, which correspond to the redox reactions observed in the cyclic voltammetry measurements. The two plateaus observed in the charge/discharge profile are very similar to those observed in Na_3_V_2_O_1.6_(PO_4_)_2_F_1.4_ and Na_3_(VO_0.5_)_2_(PO_4_)_2_F_2_. The material delivered an initial charge and discharge capacity of 146 and 117 mAh g^−1^ respectively at 1C. The Na half‐cell exhibits excellent cycling performance with a stable discharge capacity of 107 mAh g^−1^ after 900 cycles with a capacity retention of about 85% corresponding to high coulombic efficiency of 99.9% (Figure 2c). To observe the electrochemical performance of the cathode Na_3.2_Ni_0.2_V_1.8_(PO_4_)_2_F_2_O, a rate capability assessment up to a rate of 5C at room temperature was carried out. As shown in Figure 2d, the cell was cycled between 2.5 and 4.5 V versus Na^+^/Na while increasing the C rate from C/10 to 5C in a sodium half‐cell. The Na_3.2_Ni_0.2_V_1.8_(PO_4_)_2_F_2_O cathode delivered the discharge capacities of 117, 117, 116, 113, 110, and 105 mAh g^−1^ at the rates of C/10, C/5, C/3, 1C, 2C, and 5C, respectively. This result confirms that the Na_3.2_Ni_0.2_V_1.8_(PO_4_)_2_F_2_O cathode exhibits excellent rate capacity thanks to the fast Na^+^‐extraction insertion processes into the Na_3.2_Ni_0.2_V_1.8_(PO_4_)_2_F_2_O structure at investigated C rates.

**Figure 3:**
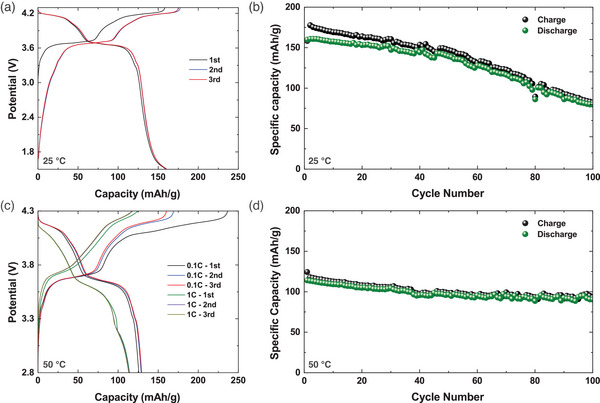
Charge/discharge curves of Na_3.2_Ni_0.2_V_1.8_(PO_4_)_2_F_2_O cathode, in a CC mode, at 0.1C and 1C rate, in the voltage range 1.5–4.3 and 2.8–4.3 V versus Na^+^/Na recorded at a) 25 °C and c) 50 °C, respectively. Half‐cell capacity as a function of cycle number at b) 25 °C and d) 50 °C.

To check the possibility of insertion of a fourth Na in Na_3.2_Ni_0.2_V_1.8_(PO_4_)_2_F_2_O framework as reported by Bianchini et al. for Na_3_V_2_(PO_4_)_2_FO_2_,^[^ ^13^ ^]^ the cell was discharged to 1.5 V and charged to 4.3 V at the rate of 0.1C at 25 °C (**Figure** **3**a). The Na_3.2_Ni_0.2_V_1.8_(PO_4_)_2_F_2_O delivers a charge capacity of 165 mAh g^−1^, which indicates that three sodium atoms have been extracted (theoretical capacity = 65 mAh g^−1^ for one sodium). However, the capacity started fading rapidly after 45 cycles which could result from side reactions within the wide voltage range (Figure 3b). We further adopted narrower voltage range (2.8–4.3 V) for cycling test to improve the capacity retention. Furthermore, we evaluated the charge/discharge performance of Na_3.2_Ni_0.2_V_1.8_(PO_4_)_2_F_2_O at 50 °C to study the tolerance of the cathode material toward elevated temperature, which is important in preventing thermal runaways that ultimately lead to fire or explosion. Figure 3c presents the first to third galvanostatic charge/discharge profiles for Na_3.2_Ni_0.2_V_1.8_O(PO_4_)_2_F_2_ compound at 0.1C and 1C for 50 °C using cut‐off voltage of 4.3 V. The Na_3.2_Ni_0.2_V_1.8_(PO_4_)_2_F_2_O samples showed good long‐term durability with the capacity loss of 0.05% per cycle on average at a 1C rate at 50 °C (Figure 3d). It should be noted that for this study we only wanted to detect the impact of potential window on the cycling stability of the developed cathode materials. Further investigation into stabilization of the cathode for the whole voltage window is needed.^[^ ^24^ ^]^

### 2.5 Full‐Cell Battery Performance

**Figure 4:**
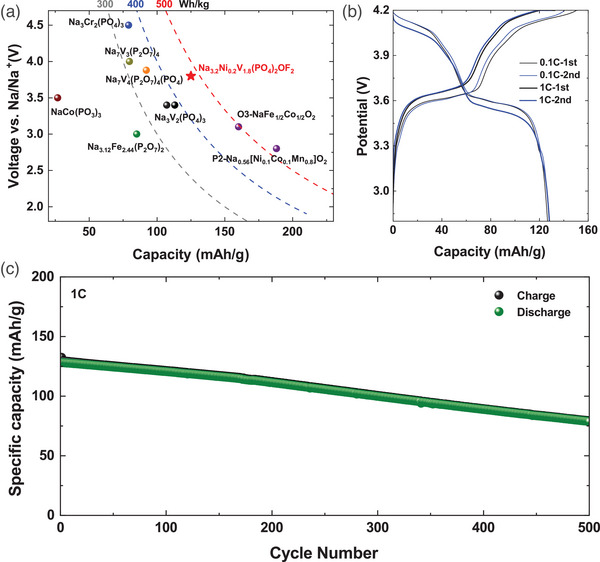
a) Comparison of voltage and specific capacity of Na_3.2_Ni_0.2_V_1.8_(PO_4_)_2_F_2_O sodium‐ion battery systems that were reported in the literature, with dotted lines indicating energy density that can be achieved at the electrode level against hard carbon anode. b) Charge/discharge curves of the Na_3.2_Ni_0.2_V_1.8_(PO_4_)_2_F_2_O | hard carbon full cell and c) its capacity evolution with cycle number at room temperature.

For practical application, it is important to demonstrate full cell performance with competitive energy densities. **Figure** **4**a summarized the nominal voltage and capacities of sodium cathodes that have been reported in literature.^[^ ^25^ ^]^ Our research team analyzed the performance of the full cell using Na_3.2_Ni_0.2_V_1.8_(PO_4_)_2_F_2_O against presodiated hard carbon anode in 1 m NaClO_4_ in ethylene carbonate–dimethyl carbonate electrolyte cycled between 2.8 and 4.2 V at 1C rate. The full cell demonstrated excellent cell performance of an initial reversible capacity of 123 mAh g^−1^ delivered at 1C (Figure 4b). The long‐term cycling performance at 1C (Figure 4c) indicates that 83 mAh g^−1^ capacity was maintained after 500 cycles, which corresponds to 85% capacity retention. The stable cycling performance can be attributed to sufficient Na^+^ in the presodiated hard carbon anode and the excellent cycling performance of the Na_3.2_Ni_0.2_V_1.8_(PO_4_)_2_F_2_O cathode material.

### 2.6 In Situ X‐Ray Powder Diffraction

**Figure 5:**
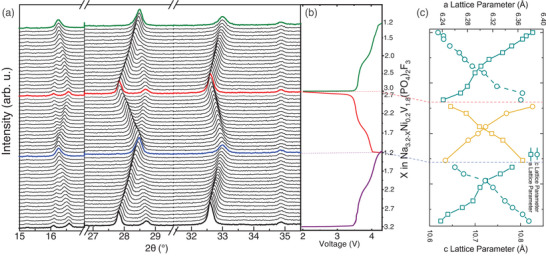
a) Operando X‐ray powder diffraction (XRD) collected during the first charge/discharge for Na_3.2_Ni_0.2_V_1.8_(PO_4_)_2_F_2_O versus Na^+^/Na under a current rate of C/10 between 2 and 4.5 V, and b) corresponding voltage profile. The diffraction results indicate a major peak shifting in situ XRD panels from 15° to 17.5°, 26.9° to 29.9°, and 32.7° to 32.9°. c) Variations of the cell parameters of Na_3.2_Ni_0.2_V_1.8_(PO_4_)_2_F_2_O during charge and discharge of the battery are shown.

To further confirm the sodium intercalation/deintercalation mechanism into/from the host structure, in situ XRD data were collected during the charge/discharge processes of Na_3.2_Ni_0.2_V_1.8_(PO_4_)_2_F_2_O | Na half‐cell in the potential range of 2–4.3 V (**Figure** **5**a,b). During the charging process, only the major peak shifting is observed (Figure 5a). The diffraction peaks (101) and (002) located at 16.16° and 16.66° merged to a single peak and returned to their original position during the discharge, revealing a reversible extraction/insertion of Na ions in the potential range of 2–4.3 V. Similar behavior was observed for the peaks (200) and (103) at 27.88° and 28.77° respectively. The peak (002) at 16.66° shifts to a lower 2*θ* angle, which corresponds to an increase of the *c* cell parameter. The peak (200) at 27.88° shifts to a higher 2*θ*, which indicates a decrease of the *a* and *b* cell parameters during charging. The reversed situation occurs during discharge, and the complete original pattern is recovered after discharging, indicating the reversible nature of the intercalation process. The cell volume was calculated using the derived lattice parameters (Figure 5c) and decreased from 432.66 to 420.75 Å^3^ during the charging process. This agrees with the extraction of sodium ions from the structure and the oxidation of vanadium cations. The XRD operando analysis clearly confirms that during the charging/discharging processes, the sodium intercalation/deintercalation in Na_3.2_Ni_0.2_V_1.8_(PO_4_)_2_F_2_O occurs through a reversible single‐phase reaction instead of a two‐phase reaction which was observed for many other phosphate‐based cathode materials.^[^ ^26^ ^]^ This result indicates the high structural stability of the Na_3.2_Ni_0.2_V_1.8_(PO_4_)_2_F_2_O during electrochemical cycling.

The measurements of galvanostatic intermittent titration (GITT) and electronic impedance spectroscopy (EIS) were performed as a function of sodium content. The EIS spectra were recorded on the half‐cell configuration of Na_3.2_Ni_0.2_V_1.8_(PO_4_)_2_F_2_O | Na at different states of charge and discharge. The selected impedance spectra at different states of charge/discharge (SOC/SOD) are shown in Figure S6 in the Supporting Information. The cell voltage decay rate was ≈2 mV h^−1^ at the end of the rest interval under the open circuit voltage (OCV) conditions, ensuring that the partial sodiation and desodiation takes place due to the applied small bias potentials during the EIS measurements. This is because, at steady state OCV condition, the cell voltage of the material lies between the oxidation and reduction states. The measured Nyquist plots of the Na_3.2_Ni_0.2_V_1.8_(PO_4_)_2_F_2_O | Na cell indicates the following components corresponding to an equivalent circuit shown in Figure S7 in the Supporting Information

- R_1_: An intercept appeared at the real part at a high‐frequency interval because of the ionic resistance of the electrolyte solution and a minor contribution of the SEI.
- R_2_: A first depressed semicircle, at the medium‐high frequencies, is due to the charge transfer resistance at the metallic sodium/electrolyte interface and the electronic conductivity of Na_3.2_Ni_0.2_V_1.8_(PO_4_)_2_F_2_O.
- R_3_: A second semicircle appeared at the medium‐low frequencies because of the charge transfer reaction at the Na_3.2_Ni_0.2_V_1.8_(PO_4_)_2_F_2_O/electrolyte interface, which has higher capacitance value than that of the first semicircle.
- The Warburg response appeared at the lower frequency interval of the spectrum.

Noting that the similar impedance spectra were appeared at other states of charge and discharge during the measurements process.

**Figure 6:**
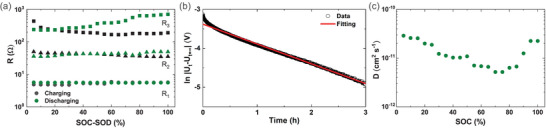
a) The ohmic (R1) and electrode/electrolyte interfacial (R2 and R3) resistances, b) fitting of depolarization cell voltage with Equation 1 to extract the relaxation time, and c) the sodium‐ion diffusivity as a function of state of charge.

The individual cell resistance components were separated by fitting the impedance spectra with the equivalent circuit displayed in Figure S7 in the Supporting Information. The ohmic resistance of the electrolyte solution (R_1_) and the interfacial charge transfer resistances (R_2_ and R_3_ for the two semicircles) are shown in **Figure** **6**a as a function of the states of charge and discharge. The EIS data comprises of seven circuit elements, which are discussed in detail in the supplementary file (Cf. the equivalent circuit model used to fit the data). It is discernible from Figure 6a, at the beginning of desodiation, the resistance of the first semicircle (R_2_) slightly decreases (up to ≈20% SOC) and, thereafter, remains almost constant with further desodiation. The initial decrease of R_2_ is assumed to be due to the change of electronic conductivity of Na_3.2_Ni_0.2_V_1.8_(PO_4_)_2_F_2_O on charging. On the other hand, the resistance of the second semicircle (R_3_) initially decreases fast with charging and thereafter, slowly decreases on further desodiation. The resistance of the lower‐frequency semicircle (R_3_) is significantly higher than that of the first semicircle (R_2_). In reality, R_2_ should not be changed with the changing of SOC as long as it is associated only with the charge transfer reaction at the sodium/electrolyte interface. However, the change of SOC/SOD could generate the mixed valent state of transition metals in the active material and induces the change of electronic conductivity which impacts the charge transfer resistance at the R3/electrolyte interface. However, the R_3_ value due to the discharge process is slightly different than the charging process and is not clearly understood. Nonetheless, R_1_ and R_2_ exhibit similar values and patterns as a function of states of charge and discharge. Recently reported data show that the charge transfer kinetics at the cathode‐electrolyte interface can be further enhanced with the increase of electronic conductivity of active materials.^[^ ^27^ ^]^ It should be noted that the electronic conductivity of Na_3.2_Ni_0.2_V_1.8_(PO_4_)_2_F_2_O is relatively low in the fully sodiated phase and increases on partial desodiation. The EIS results indicate that the interfacial charge transfer resistance at the Na_3.2_Ni_0.2_V_1.8_(PO_4_)_2_F_2_O | electrolyte interface appears to be rate‐limiting.

With the Equation (1), the relaxation times (*τ*) was extract by fitting the depolarization cell voltage of the GITT measurements at every 5% states of SOC

(1) $$\ln\;|U_{t}-U_{t\;=\;\infty }|\;=\;\ln A-\frac{t}{\tau }$$

Details of the equations and data analysis can be found in the literature.^[^ ^26^ ^]^ Here *U*_t_ and *U*_t = ∞_ are the cell voltages at times *t* and *t_∞_*, respectively and *A* is the constant term for a particular material. Noting that the slope of the plot, ln |*U_t_* − *U*_t = ∞_ | versus *t* provides the relaxation time, *τ*.

It should be noted that the origin of the exponential type of depolarization of the cell voltage is due to the sodium concentration gradient generated across the active particle during GITT measurements. In fact, the cell voltage was allowed to relax under the OCV condition leading to a steady state at each desodiation/sodiation steps. Figure 6b clearly shows that the depolarization cell voltage is well‐fitted with the Equation (1). The sodium‐ion diffusivity was derived from the obtained relaxation time which is displayed in Figure 6c as a function of the SOC. Noting that 1 µm diffusion length was considered based on the SEM microstructure. It should be noted that alternative strategies for estimating ionic diffusivity parameters from GITT measurements exist and the current approach is selected due to need of fewer unknown variables.^[^ ^28^ ^]^ Figure 6c shows two trends of ionic diffusivity behavior as a function of the SOC. Initially, the ionic diffusivity gradually decreases upon the removal of sodium up to ≈80% SOC and thereafter starts to increase gradually with the further removal of sodium to 100% SOC. Note that the diffusivity value increases to the initial value at the end of SOC. In addition, it should be noted that the diffusion mechanism of cations in crystalline materials takes place through either the interstitial space or vacancy migration, depending on local energy environment. In the interstitial mechanism, the ionic diffusivity decreases as the concentration of mobile ions decreases, whereas in the vacancy‐type mechanism, the reverse trend is observed. Therefore, the interstitial mechanism might be active up to ≈80% SOC and thereafter change to the vacancy‐type mechanism. Nonetheless, the diffusivity values fall between 5×10^−12^ and 3×10^−11^ cm^2^ s^−1^ in the whole range of the sodium concentration. The ionic diffusivity can also be a rate‐limiting factor at higher cycling rates, and micrometer‐size particles would be suitable for practical application.

## 3 Conclusion

In summary, we have successfully synthesized the sodium rich Na_3.2_Ni_0.2_V_1.8_(PO_4_)_2_F_2_O material by hydrothermal reaction with homogeneous spherical morphology. The impurity‐free stable phase is confirmed by XRD and NMR results, which includes the VO_5_F octahedra, PO_4_ tetrahedra, and bi‐octahedra (V/Ni)_2_O_10_F_2_ crystal arrangement. Half cells using Na_3.2_Ni_0.2_V_1.8_(PO_4_)_2_F_2_O as the cathode exhibited an initial capacity of 117 mAh g^−1^ at 0.1C and stable cycling performance performances up to 900 cycles with an 85% capacity retention at room temperature. The higher specific capacity and stability for these materials arises from their sodium‐rich structure, as well as co‐substitution of transition metal and fluorine in the lattice. In elevated temperature environment (50 °C), the half‐cell showed improved cycling stability, which addressed safety concerns regarding prevention of thermal runaways. Full cells with Na_3.2_Ni_0.2_V_1.8_(PO_4_)_2_F_2_O cathode and presodiated hard carbon anode achieved an initial reversible capacity of 123 mAh g^−1^ at 1C with a capacity retention of 85% after 500 cycles. Na ion diffusivity values upon initial charging/discharging are around 10^−11^ cm^2^ s^−1^, which indicated fast ionic transport properties in the NASICON‐type Na_3.2_Ni_0.2_V_1.8_(PO_4_)_2_F_2_O. Operando XRD results demonstrated reversible phase transition of the Na_3.2_Ni_0.2_V_1.8_(PO_4_)_2_F_2_O during charging/discharging. The obtained results indicate that Na_3.2_Ni_0.2_V_1.8_(PO_4_)_2_F_2_O is a promising cathode candidate for practical stable sodium‐ion batteries for both high‐power and high‐energy density applications.

## 4 Experimental Section

### Preparation

Na_3.2_Ni_0.2_V_1.8_(PO_4_)_2_F_2_O was prepared using a hydrothermal reaction by mixing stoichiometric amounts of NaF (Aldrich, ≥ 99%), NH_4_VO_3_ (Aldrich, ≥ 99.99%), NH_4_H_2_PO_4_ (Aldrich, 99.99%), Ni(CH_3_COO).4H_2_O (Aldrich, ≥ 99%), NaOH (Aldrich, ≥ 99%), and citric acid (C_6_H_8_O_7_). Here, C_6_H_8_O_7_ precursor played multiple roles in the synthesis process—chelating agent, reducing agent, and carbon source. First, NH_4_VO_3_ and citric acid with a mole ratio of 1:2 were dissolved in 40 mL of water to form a clear blue solution (solution A), indicating that V^5+^ was successfully reduced to V^3+^. Stoichiometric amounts of NaF, NaOH, and NH_4_H_2_PO_4_ were dissolved together in 40 mL of H_2_O (solution B). Solution B was then added dropwise to solution A under continuous stirring at 70 °C for 2 h or until a homogeneous solution was formed. Finally, the solution was poured into a 100 mL autoclave, which was heated at 200 °C for 20 h, then cooled down to room temperature. The resultant solution was ultrasonically treated for 30 min to increase the homogeneity of the dispersion and then was heated at 100 °C with stirring to evaporate the water. The obtained powder was then sintered at 650 °C for 12 h under argon.

### X‐Ray Powder Diffraction Measurements

To assess the purity of the Na_3.2_Ni_0.2_V_1.8_(PO_4_)_2_F_2_O sample, X‐ray Diffraction (XRD) measurements were performed. The data was collected at room temperature over the 2*θ* angular range of 10° ≤ 2*θ*  ≤ 110° with a step size of 0.01° using a Bruker D8 ADVANCE diffractometer operating with Cu‐K*α* radiation. The full pattern‐matching refinements were performed using the Jana2006 program package.^[^ ^29^ ^]^ The backgrounds were estimated by a Legendre function, and the peak shapes were described by a pseudo‐Voigt function. The Rietveld refinements were then performed using high‐resolution synchrotron XRD data collected from the 11 BM beamline of the Advanced Photon Source (*λ* = 0.1173 Å).

A specific cell with a beryllium window was used for recording operando X‐ray diffraction data in reflection mode to study the reaction mechanism of Na_3.2_Ni_0.2_V_1.8_(PO_4_)_2_F_2_O active cathode material during electrochemical cycling. The XRD patterns were recorded every hour. Diffractograms were registered during the electrochemical charge/discharge process, using Bruker D8 Discover Twin‐Twin with an advanced diffractometer in Bragg–Brentano geometry with Cu‐K*α* radiation (*λ*  =  1.540, 60 Å, 15° ≤ 2*θ* ≤ 40°) and a Bio‐Logic VMP3 potentiostat at a rate of C/20.

### Electron Diffraction Investigations

Selected area electron diffraction and high‐resolution transmission electron microscopy (HRTEM) were performed in a JEOL 3000 FEG electron microscope, fitted with a double tilting goniometer stage (±22°, ±22°). The local composition was analyzed by energy‐dispersive X‐ray spectroscopy with an Oxford INCA analyzer system attached to the above‐mentioned microscope. Simulated HRTEM images were calculated by the multislice method using the MacTempas software package.

### Electrochemical Measurements

Positive electrodes were made from a mixture of Na_3.2_Ni_0.2_V_1.8_(PO_4_)_2_F_2_O powder, acetylene black, and polyvinylidene fluoride in a weight ratio of 80:10:10. The resulting electrode films were cut into round discs (*Φ* = 14 mm) and dried at 120 °C for 12 h under vacuum. 100 µL of 1 m NaPF_6_ dissolved in ethylene carbonate (EC) and propylene carbonate (PC) (v:v, 1:1) was used as electrolyte. The CR2032 coin cells were assembled against Na metal in an Ar‐filled glove box, with the glass fiber (Whatman Grade GF/A, *Φ* = 20 mm) as a separator. Galvanostatic charge/discharge tests were performed on an Arbin and Maccor battery testing systems under various current densities. Hard carbon anodes are presodiated in half‐cell configuration against Na metal (0.1C for one cycle, then discharged to 100%), washed with PC, and finally dried before further use. Full cells are made by pairing cathodes with presodiated hard carbon anodes with 1 m NaClO_4_ in PC with 5 vol% fluoroethylene carbonate as the electrolyte. Full cells are cycled between 2.8 and 4.2 V at 0.1C (1C = 128 mA g^−1^) for five cycles, then at 1C for extensive cycling.

### Impedance Spectroscopy Measurements

In situ electrochemical impedance spectroscopy (EIS) measurements were performed to determine the interfacial charge transfer resistance as a function of sodium content within the cathode. The cell voltage relaxation process was applied to evaluate ionic diffusivity. In the formation cycle, the cell was charged and discharged to form a stable SEI layer, and then a charging/discharging current equivalent to a C/20 rate was applied using a Solartron battery cycler (1470 E). The current was applied for 1 h to attain a certain state of charge/discharge. After partial sodiation/desodiation, the cell voltage was relaxed for at least 3 h under the open‐circuit voltage (OCV) condition to reach the steady state in which the voltage decay is less than 2 mV h^−1^ at the end of the time interval. Thereafter, EIS measurement was performed in the frequency range of 5 MHz to 0.5 Hz using a sinusoidal voltage amplitude of 10 mV. The measurement procedure was repeated stepwise to cover all states of charge/discharge. The depolarized cell voltage was fitted to evaluate the ionic diffusivity, and the measured EIS spectra were fitted using an equivalent circuit model.

### NMR Measurements

All experiments were performed using a Varian 300 MHz Direct Drive Spectrometer with an operating frequency of 301.4 MHz at a field of 7.05T. A 3.2 mm MAS Chemagnetics broadband probe was utilized, and all samples were packed into 3.2 mm thin‐walled zirconia rotors with Vespel end caps and drive tips. Single‐pulse and Spin Echo experiments were performed to obtain ^7^Li, ^23^Na, and ^31^P spectra at a spinning speed of 19 kHz, and ^19^F spectra at a speed of 15 kHz.

## Conflict of Interest

The authors declare no conflict of interest.

## Supporting information

Supporting Information

## Data Availability

The data that support the findings of this study are available from the corresponding author upon reasonable request.
