# Supplementary material for: Sodium Rich Vanadium Oxy‐Fluorophosphate – Na3.2Ni0.2V1.8(PO4)2F2O – as Advanced Cathode for Sodium Ion Batteries
Source: Adv Sci (Weinh). 2023 May 18;10(22):2301091. doi: 10.1002/advs.202301091 (PMC10401166; doi:10.1002/advs.202301091)
Supplement: Supplementary file 1 — Supporting Information [file ADVS-10-2301091-s001.pdf]

ADVANCED SCIENCE
Open Access

Supporting Information

for *Adv. Sci.*, DOI 10.1002/advs.202301091

Sodium Rich Vanadium Oxy-Fluorophosphate – $Na_{3.2}Ni_{0.2}V_{1.8}(PO_4)_2F_2O$ – as Advanced Cathode for Sodium Ion Batteries

Rachid Essehli, Hamdi Ben Yahia, Ruhul Amin, Mengya Li, Daniel Morales, Steven G. Greenbaum, Ali Abouimrane, Anand Parejiya, Abdelfattah Mahmoud, Khalid Boulahya, Marm Dixit and Ilias Belharouak**

Supporting Information

Sodium Rich Vanadium Oxy-fluorophosphate - $Na_{3.2}Ni_{0.2}V_{1.8}(PO_4)_2F_2O$ - as Advanced Cathode for Sodium Ion Batteries[1]

Rachid Essehli,[a,]* *Hamdi Ben Yahia,*[b] *Ruhul Amin,*[a] *Mengya Li,*[a] *Steven G. Greenbaum,*[c] *Ali Abouimrane,*[a] *Anand Parejiya,*[a] *Abdelfattah Mahmoud,*[d] *Khalid Boulahya,*[e] *Marm Dixit,*[a] *and Ilias Belharouak*[a,]*

[a] Electrification and Energy Infrastructures Division, Oak Ridge National Laboratory, Oak Ridge, TN 37831 USA

[b] Qatar Environment and Energy Research Institute Hamad Bin Khalifa University, Qatar Foundation, Doha, 34110 Qatar

[c] Department of Physics & Astronomy, Hunter College of the City University of New York, New York, New York 10065, United States

[d] GREENMAT, CESAM Research Unit, University of Liège, Department of Chemistry, Liège 4000, Belgium

[e] Departamento de Química Inorgánica Facultad de Químicas Universidad Complutense, 28040 Madrid Spain

E-mail: belharouaki@ornl.gov; essehlir@ornl.gov

Keywords: Sodium-ion battery, Vanadium Oxy-fluorophosphate, Cathode, Batteries, Energy storage

---

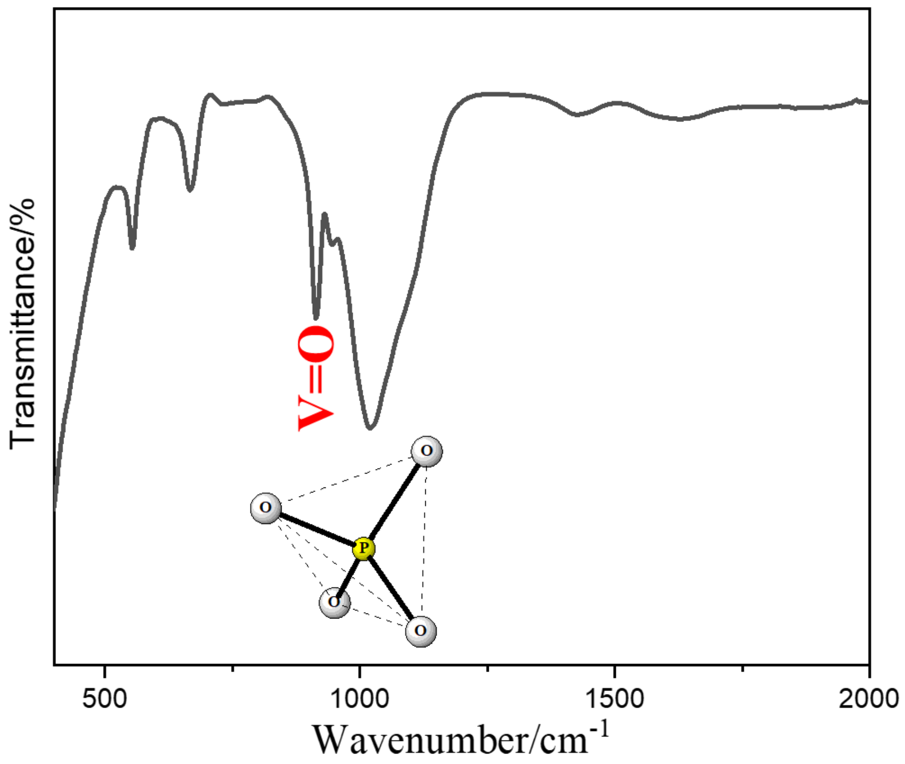

Figure S1. The Fourier transform infrared spectroscopy spectrum of $Na_{3.2}Ni_{0.2}V_{1.8}(PO_4)_2F_2O$ composite in the range of 400–2,000 wavenumbers.

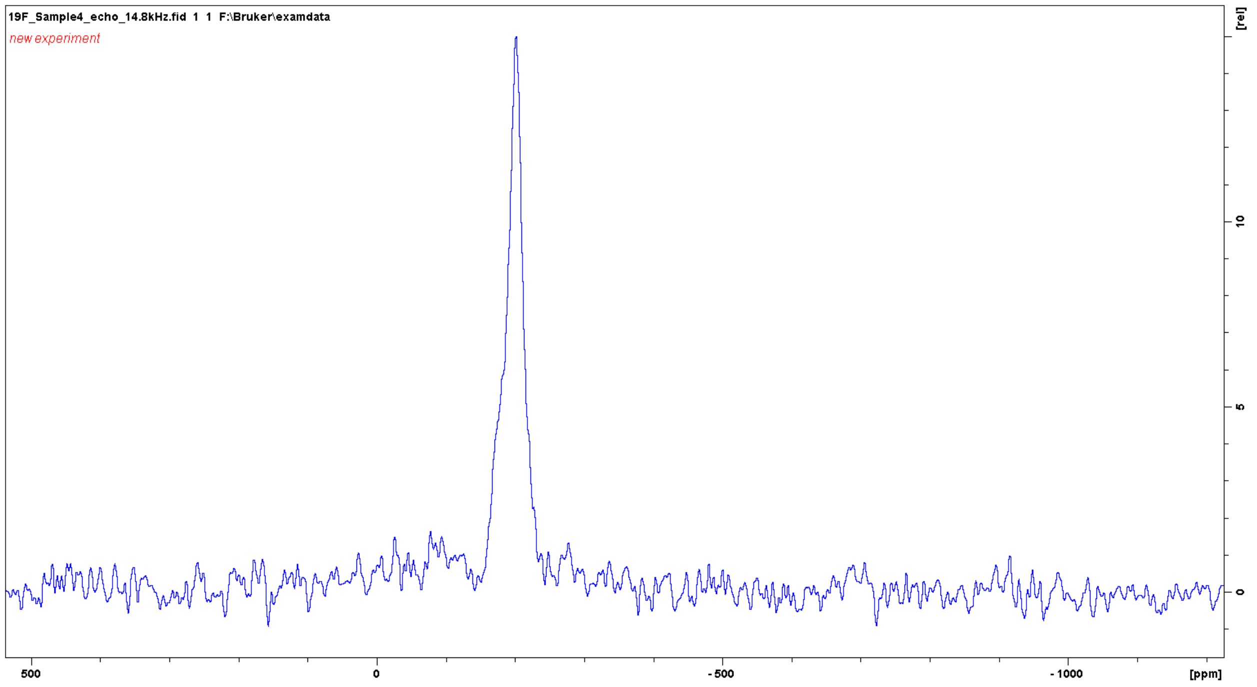

Figure S2. Nuclear Magnetic Resonance spin echo spectrum of ^{19}F in $Na_{3.2}Ni_{0.2}V_{1.8}(PO_4)_2F_2O$.

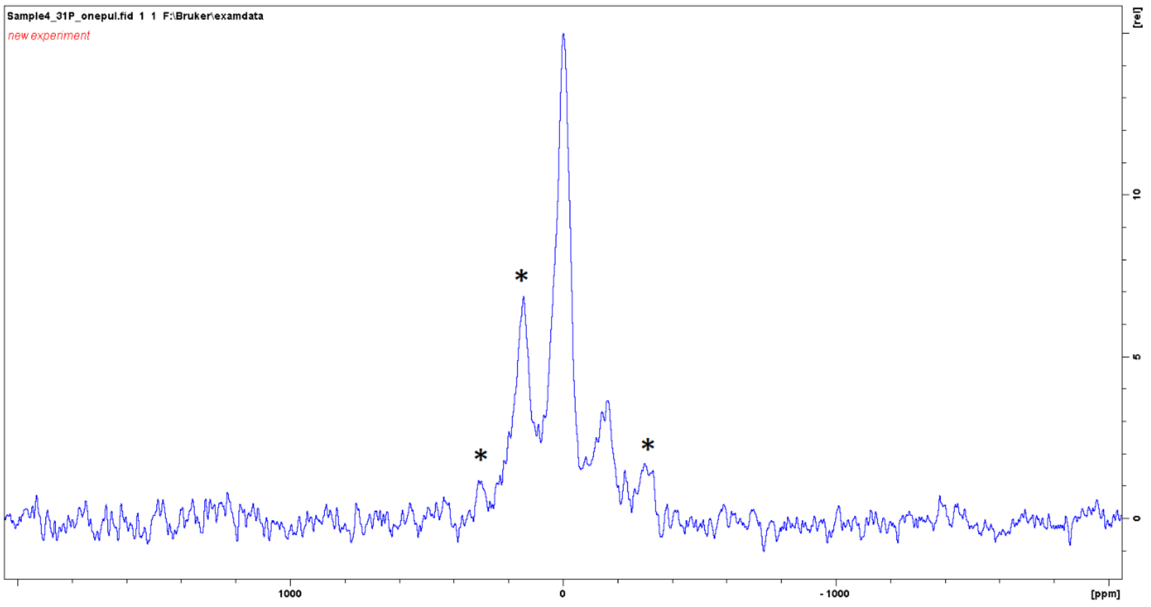

Figure S3. Nuclear Magnetic Resonance spin echo spectrum ^{31}P in $Na_{3.2}Ni_{0.2}V_{1.8}(PO_4)_2F_2O$ near 0 ppm. Peaks with an asterisk denote spinning sidebands.

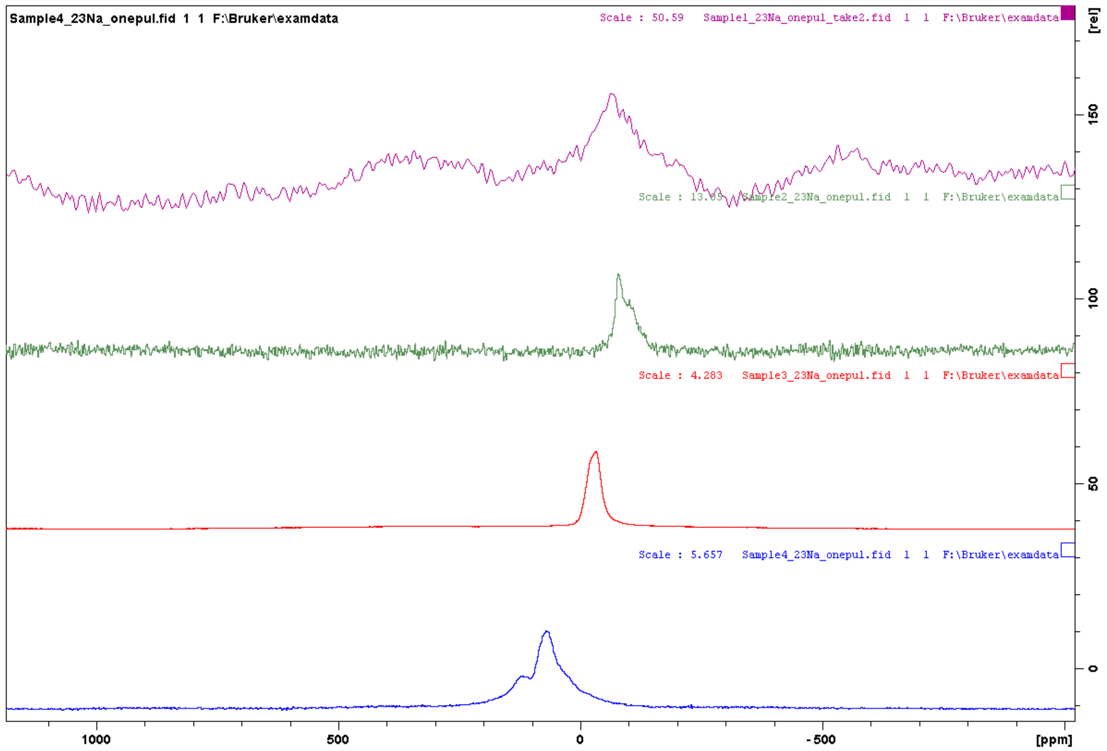

Figure S4. [23]Na Nuclear Magnetic Resonance spin echo spectra $Na_{3.2}Ni_{0.2}V_{1.8}(PO_4)_2F_2O$ (red).

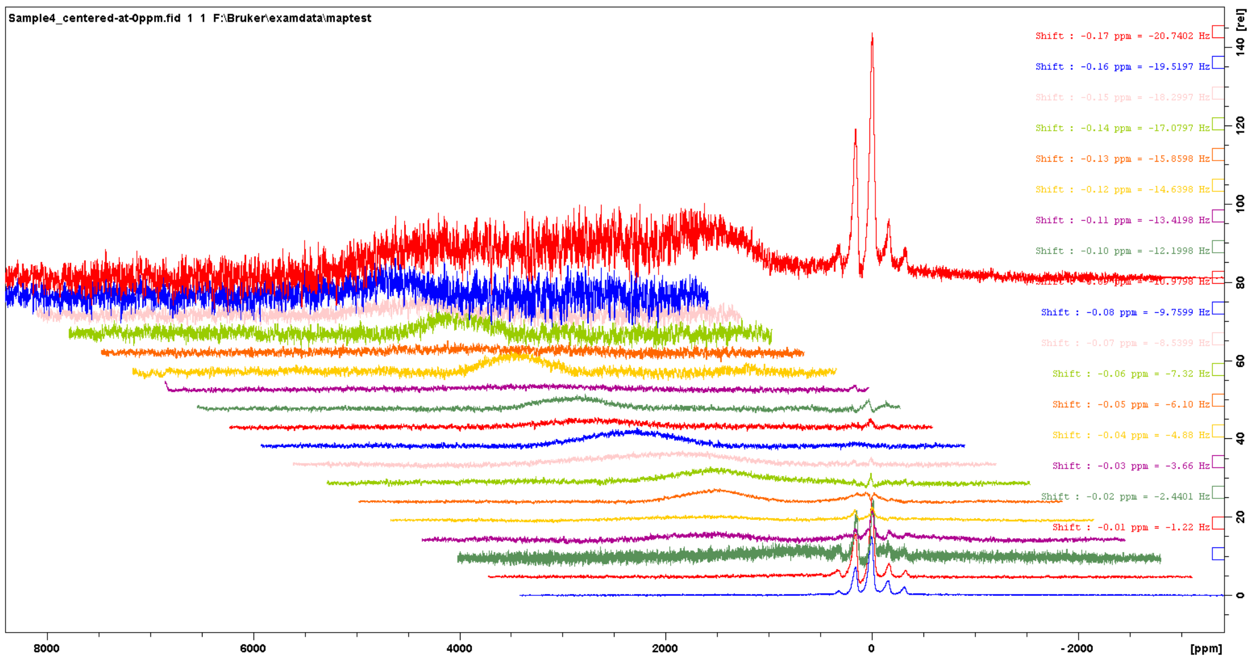

Figure S5. The Nuclear Magnetic Resonance spectra for sodium in $Na_{3.2}Ni_{0.2}V_{1.8}(PO_4)_2F_2O$(red). The carrier frequency was shifted in intervals of 37.8 kHz to resolve the broad components from 1,000 to 4,000 ppm.

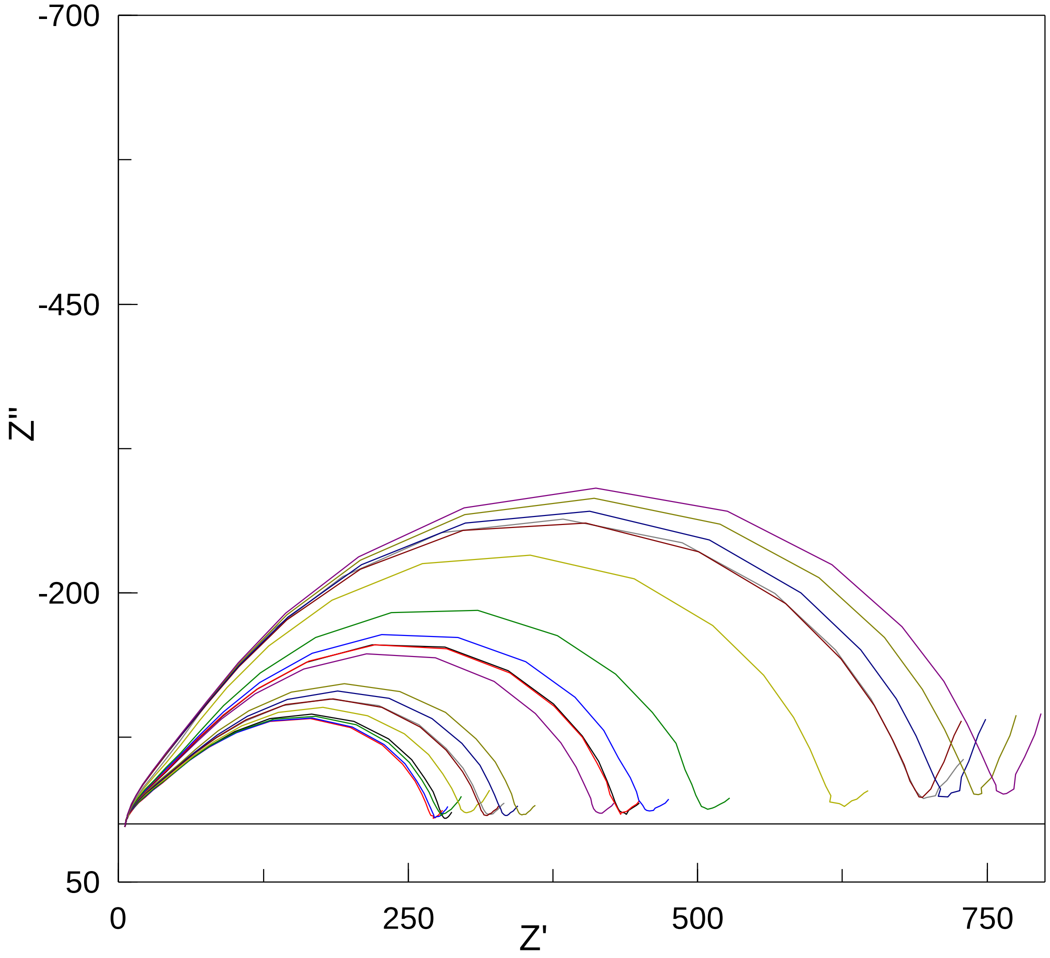

Figure S6. Impedance spectra as a function of state of charge at ambient conditions, consisting of high-frequency intercept, with two semicircles in the medium frequency regime followed by Warburg diffusion spike.

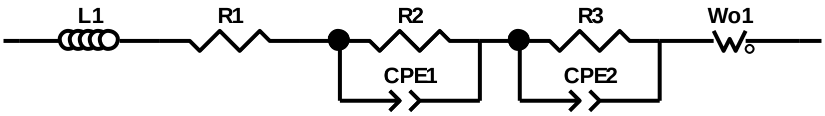

Figure S7. Equivalent circuit model is used to fit the impedance spectra. L1 is the inductance due to wire and other parts of the cell. R1 is the series resistance due to the electrolyte. R2 is the

charge transfer interfacial resistance due to the anode/electrolyte interface. R3 is the charge transfer interfacial resistance due to the cathode/electrolyte interface. Wo1 is the Warburg part due to the diffusion. CPE is the constant phase element.

Table S1. Structural Parameters Obtained from Rietveld Refinement of Synchrotron Radiation Diffraction Data of $Na_3V_2(PO_4)_2F_3$ at T = 400 K from Chem. Mater. (2014) 26, 4238-4247.

Atom	Wyck.	occupancy	x/a	y/b	z/c	Biso
Na(1)	8h	0.45(3)	0.277(1)	0.277(1)	0	2.3(5)
Na(2)	16l	0.15(2)	0.409(5)	0.225(4)	0	2.6(9
V	4e	1	0	0	0.1841(2)	0.67(6)
P	4d	1	1/2	0	1/4	0.75(8)
O	16n	1	0.3087(6)	0	0.1646(5)	1.1(1)
F(1)	2a	1	0	0	0	1.3(2)
F(2)	4e	1	0	0	0.3672(8)	1.7(2)

Table S2. EDS Elemental Quantification

Element	[wt.%]	[norm. wt.%]	[norm. at.%]
Phosphourous	11.4	15.71	11.18
Sodium	11.74	16.18	15.51
Vanadium	15.24	21.01	9.09
Oxygen	19.31	26.62	36.66
Flourine	6.72	9.27	10.75
Nickel	1.86	2.56	0.96
Carbon	6.27	8.65	15.86
Sum	72.55	100	100.0
